# ROX index versus HACOR scale in predicting success and failure of high-flow nasal cannula in the emergency department for patients with acute hypoxemic respiratory failure: a prospective observational study

**DOI:** 10.1186/s12245-023-00477-1

**Published:** 2023-01-10

**Authors:** Nattakarn Praphruetkit, Natyada Boonchana, Apichaya Monsomboon, Onlak Ruangsomboon

**Affiliations:** Department of Emergency Medicine, Faculty of Medicine, Siriraj Hospital, Mahidol University, 2 Wanglang Road, Bangkoknoi, Bangkok, 10700 Thailand

**Keywords:** High-flow nasal cannula, Nasal high flow, ROX index, HACOR scale

## Abstract

**Background:**

High-flow nasal cannula has been a promising initial respiratory support measure for patients with acute hypoxemic respiratory failure (AHRF) in the emergency department (ED). However, delayed detection of HFNC failure is associated with increased mortality. The ROX index is a tool that can help predict HFNC success. Nonetheless, its utility in ED patients is limited, and no studies have compared it with the HACOR scale, another tool that may be as accurate in predicting HFNC failure. Therefore, we aimed to compare the prognostic utility of the ROX index and the HACOR scale in emergency AHRF patients.

**Methods:**

This prospective observational study was conducted at the ED of Siriraj Hospital, Thailand, between August 2018 and February 2020. Adult patients with AHRF requiring HFNC in the ED were included. The ROX index and the HACOR scale were measured at 1, 2, and 6 h after HFNC initiation. The primary outcome was HFNC success, defined as no intolerance or escalation towards mechanical ventilation or non-invasive ventilation within 48 h.

**Results:**

A total of 75 patients were enrolled; 52 (69.3%) had a successful treatment. The ROX index was higher in the success group, while the HACOR scale was lower at all timepoints. The ROX index yielded generally higher discrimination capacity based on the area under the receiver operating characteristic curve (AUROC) than the HACOR scale [AUROC at 1, 2, and 6 h = 0.815, 0.784, 0.853 for ROX in predicting HFNC success and 0.733, 0.690, and 0.764 for HACOR in predicting HFNC failure]. The ROX index measured at 6 h at the cut-point of 4.88 had 92.98% sensitivity, 61.11% specificity, 88.33% positive predictive value, and 73.33% negative predictive value with a diagnostic accuracy of 85.33%.

**Conclusion:**

The ROX index had superior prognostic utility in predicting HFNC outcome (success/failure) compared to the HACOR scale in patients with AHRF in the ED setting. Moreover, it is less complex and more efficient to be employed at bedside. Therefore, the ROX index is a more appropriate tool to guide further management and potential escalation therapy for AHRF patients with HFNC therapy initiated in the ED.

**Supplementary Information:**

The online version contains supplementary material available at 10.1186/s12245-023-00477-1.

## Background

Acute hypoxemic respiratory failure (AHRF) is a common and life-threatening presenting symptom to the emergency department (ED) [1]. Oxygen therapy is one of the most important initial treatment for AHRF [2]. It can be delivered via conventional technique through standard nasal cannula or facemask. However, such conventional oxygen delivering methods have several limitations including flow limitation, a varying fraction of inspired oxygen (FiO2), and insufficient heating and humidification [3, 4]. High-flow nasal cannula (HFNC) therapy is a novel device that can deliver the flow rate up to 60 L/min in adults and 100% heated and humidified oxygen via a large-bore nasal cannula [3, 4]. It has been proven to effectively improve oxygenation, decrease work of breathing, and lower the rate of escalation to invasive ventilation for patients with AHRF [5–7]. The efficacy of HFNC has also been demonstrated in the ED settings [8–10]. However, HFNC therapy needs to be used with caution, especially in critically ill patients, because delayed intubation in patients with failed HFNC treatment may increase mortality [11, 12]. Therefore, identifying factors and developing indices or scales that can predict the success or failure of HFNC are essential to reduce such delayed intubation and possibly prevent mortality. Although previous studies have demonstrated that severity scores in critically ill patients, such as sequential organ assessment (SOFA), simplified acute physiology II (SAP II), and acute physiology and chronic health evaluation II (APACHE II) score, were significant predictors of HFNC failure [13–16], these scores are complex and inconvenient to be employed as a bedside tool in the ED.

The ROX (Respiratory rate OXygenation) index, a ratio of oxygen saturation (SpO_2_)/FiO_2_ to respiratory rate, has been a known strong predictor of HFNC success [17, 18]. However, although it has been externally validated in many settings, most studies were still based in the intensive care units (ICU), with limited studies validating the utility of the ROX index in the ED, where patient characteristics and acuity may differ. On the other hand, the HACOR scale comprising of *H*eart rate, *A*cidosis, *C*onsciousness (defined by the Glasgow Coma Scale [GCS] score), *O*xygenation, and *R*espiratory rate (HACOR) is a score initially developed as a tool to predict non-invasive ventilation (NIV) failure [19]. Because NIV and HFNC both aim to prevent invasive ventilation, some studies have validated the HACOR scale for predicting HFNC failure, with most results showing favorable discrimination indices [20–22]. However, all studies were performed in the ICU, and only one study directly compared the two potential predictors [22]. Nonetheless, the study was conducted in a different patient population and setting, so their results may not be applicable to ED AHRF patients. We hypothesized that the HACOR scale might also have similar or superior prognostic utility for ED AHRF patients as the ROX index. Therefore, this study was conducted to assess the utility of the ROX index compared to the HACOR scale in predicting the success and failure of HFNC in AHRF patients in the ED.

## Materials and methods

### Study design and setting

This prospective observational study was conducted between August 12, 2018, and February 23, 2020, at the ED of Siriraj Hospital, the largest tertiary university hospital in Bangkok, Thailand, with over 2200 inpatient beds. The ED accommodates over 18,000 annual high-acuity visits triaged as level 1 or 2 based on the Emergency Severity Index (ESI) criteria [23]. The study was approved by Siriraj Institutional Review Board (certificate no. 224/2018). Written informed consent was obtained from all participants or their next of kin by the study investigators prior to the study inclusion. Standard treatment with NIV or HFNC was given to those who declined to participate at the attending physician’s discretion.

### Participants

Adult patients over 18 years of age diagnosed with AHRF and determined by the attending ED physicians to require supportive oxygen therapy via HFNC were included. AHRF requiring HFNC was determined when all of the following criteria were met; a respiratory rate of more than 24 breaths/min, the presence of accessory muscle use, room air SpO_2_ of equal to or less than 90%, and the need for oxygen supplement via face mask > 9 L/min to maintain SpO_2_ > 92% [24–26]. Exclusion criteria were immediate cardiac or respiratory arrest or respiratory failure (respiratory rate > 35 breaths/min or SpO_2_ < 90% despite oxygen supplement via facemask > 9 L/min) requiring immediate mechanical ventilation, GCS < 12, hemodynamic instability (systolic blood pressure < 90 mmHg or mean arterial pressure < 65 mmHg), severe respiratory acidosis (pH < 7.3 and arterial pressure of carbon dioxide > 50 mmHg) or suspected hypercapnic respiratory failure, pneumothorax, and do-not-intubate status.

### Study process and data collection

Patients visiting the ED with AHRF were consecutively assessed for eligibility. After ED arrival, eligible patients first received oxygen supplement via conventional technique and standard medical treatment as considered appropriate by the attending ED physician. They were then re-evaluated at 10 min and recruited if they were still eligible and did not meet any exclusion criteria. After informed consent was obtained, HFNC (AIRVO®2; Fisher & Paykel, Auckland, New Zealand) was applied at an initial flow rate of 30 L/min and FiO_2_ of 0.5. The flow could be adjusted up to 60 L/min as tolerated by the participants with FiO_2_ adjusted to maintain SpO_2_ of more than 94%. HFNC was terminated, and respiratory support was escalated to either NIV or mechanical ventilation if one of the following termination criteria, adopted from the British Thoracic Society’s guideline [27], was met; worsening dyspnea or hypoxemia (respiratory rate > 40 breaths/min or SpO_2_ < 90% despite maximum FiO_2_), worsening hypercapnia (increased PaCO_2_ or pH < 7.30 despite maximum standard medical care), deterioration of consciousness (GCS < 12 or decrease > 2 from baseline), severe hemodynamic instability (norepinephrine > 0.1 μg/kg/min or dopamine > 20 mcg/kg/min), and at the physician’s discretion. Other treatment was given as appropriate under the discretion of the treating physician.

Demographic data, including age, sex, and underlying diseases, were recorded. Respiratory and physiologic parameters (respiratory rate, signs of respiratory distress, pulse rate, blood pressure, SpO_2_, and GCS) and arterial gas results were recorded at initial ED presentation, 0, 1, 2, and 6 h after the initiation of HFNC. Participants’ diagnoses, management, and outcomes were also recorded.

### Study parameters and outcomes

At 1, 2, and 6 h after HFNC initiation, two parameters were assessed for their utility in predicting HFNC success: the ROX index and SpO_2_/FiO_2_ (SF) ratio. The ROX index was calculated from the ratio of SF to respiratory rate [17]. At the same timepoints, two parameters were evaluated for their predictive ability of HFNC failure: respiratory rate and the HACOR scale. The HACOR scale (0–25 points) consists of five components with weighted score points [19]. The components are pulse rate (0–1 point), pH (0–4 points), GCS (0–10 points), PaO_2_/FiO_2_ (0–6 points), and respiratory rate (0–4 points).

Successful HFNC was defined as no intolerance requiring equipment removal and no escalation to invasive or non-invasive ventilation within 48 h after HFNC application. The primary aim of the study was to assess the predictive accuracy of the ROX index and the HACOR scale in predicting HFNC success and failure, respectively. In addition, the components of the ROX index, namely SF ratio and respiratory rate, were also individually evaluated for their prognostic utility. This was performed to explore the components contributing the most to the prognostic utility of the ROX index. Moreover, we also explored the potential prognostic utility of pulse rate as an exploratory analysis since it is also one of the components of the HACOR scale, which is a known strong predictor of HFNC failure [28, 29].

### Statistical analyses

The initial sample size was calculated based on an expected sensitivity and specificity of 70% and 72%, respectively, for the ROX index at the recommended cut-point of 4.88 in predicting successful HFNC [17]. We estimated that the ROX index would have 70 ± 9% accuracy in determining HFNC success; thus, a sample size of 100 participants was required. As for the HACOR scale, we estimated that the sample size required would be equal to or lower than those for the ROX index, as we hypothesized that the HACOR scale would yield similar accuracy with a slightly higher standard error than that of the ROX index. However, we had to discontinue the trial prematurely because of the COVID-19 outbreak causing resource limitations after 75 participants were recruited.

Descriptive statistics were employed to present patient demographics, physiologic variables, and outcomes. These characteristics were compared between HFNC success and failure groups using the independent *t*-test or the Mann–Whitney *U* test for normally distributed and non-normally distributed continuous data, respectively, and the chi-squared or Fisher’s exact test for categorical data. The accuracy of the parameters in predicting the success or failure of HFNC was analyzed by using the receiver operating characteristic (ROC) curves and the area under the curves (AUROC) and its 95% confidence interval (CI). We further explored the AUROC for the subgroup with and without pneumonia as an exploratory analysis. Sensitivity, specificity, positive and negative predictive value (PPV and NPV), positive and negative likelihood ratios (LR + and LR −), and accuracy of the ROX index at the recommended cut-point (4.88) and the optimal cut-point from the data (the point from which the highest sensitivity and specificity were derived) were analyzed and presented. Moreover, we performed the log-rank test of the survival function contrasting the ROX index at the cut-point of 4.88 at all timepoints with HFNC failure as the outcome. We also performed univariate and multivariate logistic regression analyses to identify independent predictors of HFNC success. Age, sex, and underlying respiratory diseases were determined a priori as potential associating variables with HFNC success to be adjusted for in the multivariate logistic regression models. Only one parameter value at one timepoint was evaluated in each multivariate regression model to avoid multicollinearity.

A *p*-value of less than 0.05 was considered statistically significant. All statistical analyses were performed using SPSS 18 (SPSS Inc., Chicago, Illinois) except for sensitivity and specificity, LR + , LR − , NPV, PPV, and accuracy, which were calculated using MedCalc for Windows version 19 (MedCalc statistical software, Mariakerke, Belgium).

## Results

**Fig. 1:**
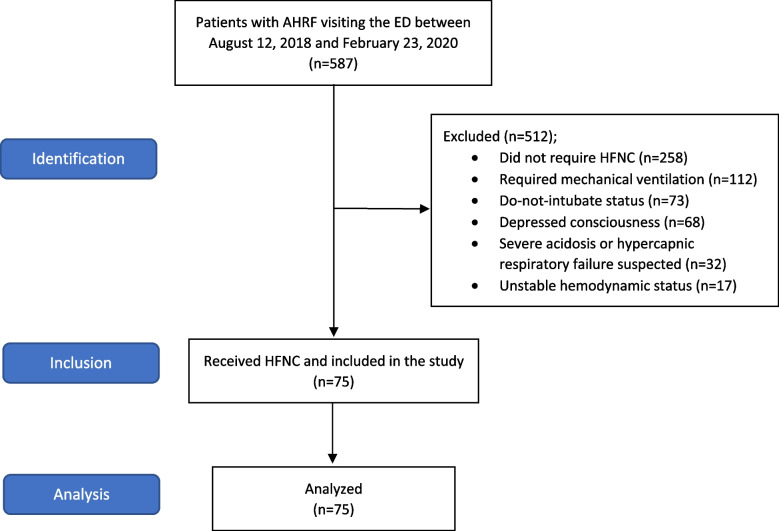
Study flow. AHRF, acute hypoxemic respiratory failure; ED, Emergency Department; HFNC, high-flow nasal cannula

Between August 12, 2018, and February 23, 2020, a total of 587 patients visited the ED with AHRF. Of these, 258 did not require HFNC, 112 required immediate mechanical ventilation, 73 had do-not-intubate status, 68 had depressed GCS, 32 had either severe acidosis or suspected hypercapnic respiratory failure, and 17 had unstable hemodynamic status. Consequently, a total of 75 patients received HFNC and were included (Fig. 1). Their characteristics are shown in Table 1. Of all included patients, 26 were male (34.5%), and their mean age was 69.12 ± 17.69 years. Twenty-three patients (30.7%) had met at least one criteria of HFNC failure, while 52 (69.3%) were successfully treated with HFNC. Baseline demographics and physiologic variables were similar between those with HFNC success and failure status except for higher initial pulse rate and respiratory rate in the failure group (*p* = 0.003 and 0.026, respectively). Primary diagnoses were similar despite a trend of higher pneumonia rate in the failure group and a higher proportion of cardiogenic pulmonary edema patients in the success group. Hospital length of stay and mortality rate was significantly higher in the failure group (*p* = 0.047 and 0.001, respectively).

**Table 1:** Patient characteristics by high-flow nasal cannula success status

	**Success (*n* = 52)**	**Failure (*n* = 23)**	***p*-value**
Male sex	17 (32.7)	9 (39.1)	0.589
Age, years	69.6 ± 17.4	68.0 ± 18.6	0.718
Underlying diseases			
Respiratory disease	43 (82.7)	17 (73.9)	0.273
Cardiovascular disease	36 (69.2)	18 (78.3)	0.422
Diabetes mellitus	28 (53.8)	12 (52.2)	0.894
Hypertension	14 (26.9)	12 (52.2)	0.034
Chronic kidney disease	40 (76.9)	19 (82.6)	0.579
Initial vital signs			
Systolic blood pressure	148.0 ± 26.1	139.7 ± 30.4	0.233
Diastolic blood pressure	75.6 ± 15.2	77.5 ± 18.0	0.634
Pulse rate	95.2 ± 21.4	110.7 ± 18.2	0.003
Respiratory rate	31.7 ± 4.6	34.6 ± 6.0	0.026
Pulse oximetry	85.0 ± 6.2	86.9 ± 5.9	0.199
Glasgow Coma scale score (median, IQR)	15, 0	15, 0	0.582
Initial arterial gas			
pH	7.43 ± 0.10	7.42 ± 0.03	0.735
Arterial pressure of oxygen	87.0 ± 32.27	85.67 ± 34.79	0.940
Arterial pressure of carbon dioxide	32.81 ± 8.26	32.40 ± 6.31	0.911
HFNC settings			
Flow (L/min)	36.06 ± 7.63	39.13 ± 8.35	0.122
Fraction of inspired oxygen	0.65 ± 0.14	0.64 ± 0.13	0.862
HFNC treatment duration, hour (median, IQR)	19.77, 54.27	10.0, 26.84	0.017
Diagnosis			
Pneumonia	17 (32.7)	13 (56.5)	0.076
Cardiogenic pulmonary edema	24 (46.2)	5 (21.7)	0.063
Others	11 (21.1)	5 (21.7)	0.503
Type of HFNC failure			
Endotracheal intubation	NA	16 (69.6)	NA
Non-invasive ventilation	NA	4 (17.4)	NA
Intolerance	NA	3 (13.0)	NA
Co-treatment			
Diuretics	32 (61.5)	5 (21.7)	0.001
Antibiotics	34 (65.4)	20 (87.0)	0.055
Bronchodilators	15 (28.8)	11 (47.8)	0.111
Mortality rate	3 (5.8)	7 (30.4)	0.001
ED length of stay, hour	14.50, 13.04	11.93, 12.20	0.075
Hospital length of stay, day	8.91, 12.87	15.59, 24.97	0.047

Data are presented as frequency (percentage), mean ± standard deviation or median, interquartile range (IQR) as appropriate

*Abbreviations*: *HFNC* High-flow nasal cannula, *ED* Emergency department

All the four potential predictors of HFNC outcome were mostly different compared between the success and the failure groups at all timepoints. The ROX index was generally increasing over time and was significantly lower in the failure than the success group at all timepoints (Table 2). On the other hand, the HACOR scale decreased over time and was significantly higher in the failure groups (Table 2). SF ratio changed and differed between the groups in the same pattern as the ROX index, while respiratory rate followed the pattern of the HACOR scale. Nonetheless, the between-group differences were more prominent at later timepoints for these two parameters, as opposed to the HACOR scale, whose difference was most notable on the first assessment (Table 2).

**Table 2:** Descriptive statistics of potential predictors of success and failure of high-flow nasal cannula

	Time (h)	Patients remaining on HFNC	Success	Patients remaining on HFNC	Failure	*p*-value
ROX index	1	52	6.99 ± 2.14	23	5.66 ± 2.18	0.024
	2	52	7.69 ± 2.40	21	6.17 ± 2.37	0.021
	6	47	8.28 ± 2.66	17	6.68 ± 3.00	0.046
HACOR scale	1	52	2.61 ± 2.23	23	4.50 ± 2.77	0.015
	2	52	2.21 ± 2.23	21	4.11 ± 2.87	0.017
	6	47	1.78 ± 1.90	17	3.67 ± 2.84	0.047
Respiratory rate (breaths/min)	1	52	26.10 ± 4.69	23	30.13 ± 5.90	0.022
	2	52	25.15 ± 3.93	21	29.18 ± 5.88	0.006
	6	47	25.42 ± 3.36	17	29.41 ± 5.60	0.001
SpO_2_/FiO_2_ ratio	1	52	179.12 ± 45.02	23	155.03 ± 43.56	0.052
	2	52	191.31 ± 51.19	21	161.03 ± 43.87	0.020
	6	47	210.11 ± 55.96	17	157.87 ± 38.34	0.001
Pulse rate (beats/min)	1	52	90.51 ± 19.44	23	101.78 ± 19.80	0.036
	2	52	90.30 ± 19.99	21	101.29 ± 23.04	0.059
	6	47	88.98 ± 18.46	17	109.17 ± 22.54	0.002

*Abbreviations*: *SpO*_*2*_ Pulse oximetry, *FiO*_*2*_ Fraction of inspired oxygen

**Fig. 2:**
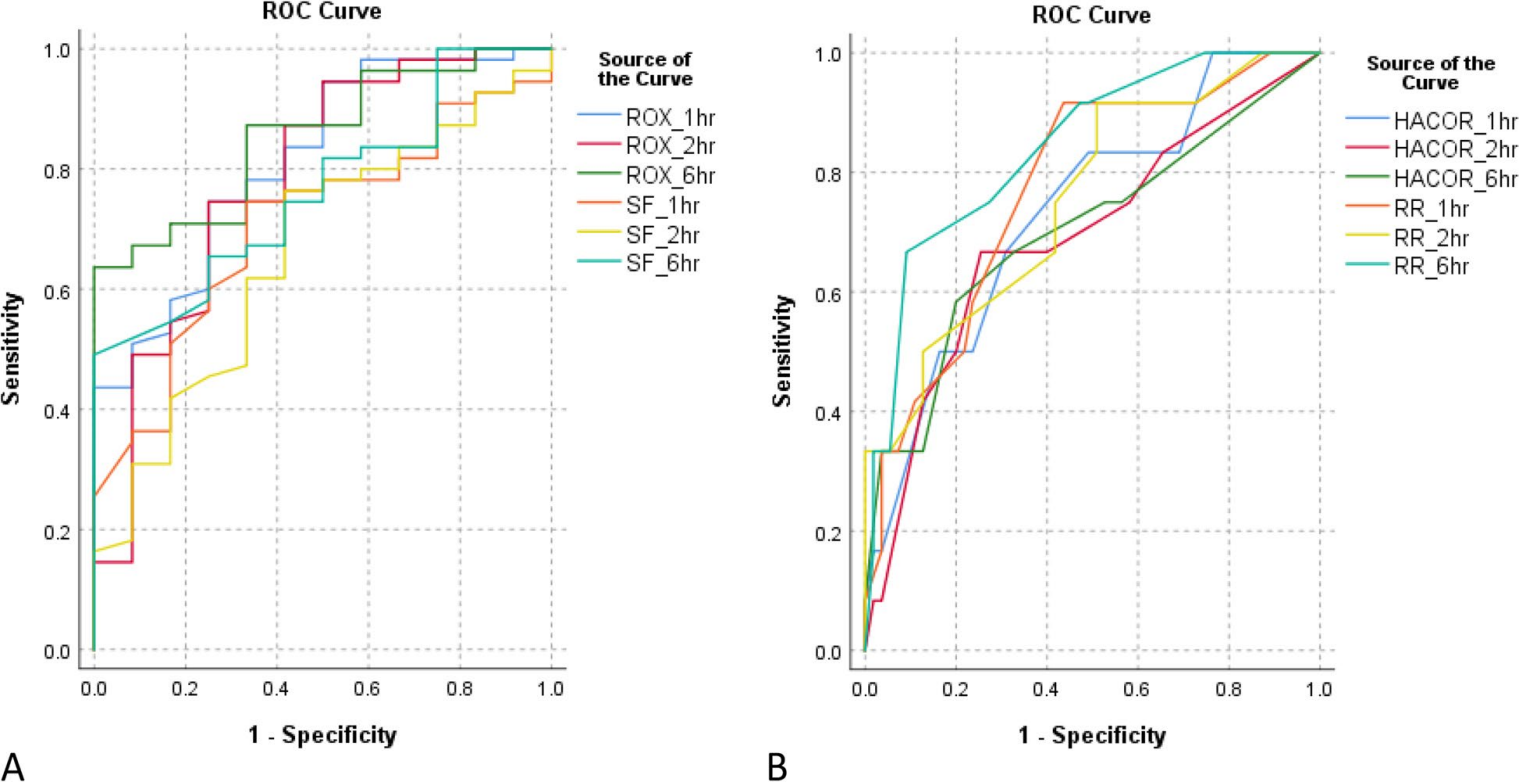
Receiver operating characteristic curves of potential predictors of high-flow nasal cannula success (**A**) and failure (**B**) at different timepoints. SF, pulse oximetry/fraction of inspired oxygen ratio; RR, respiratory rate

The ROC curves of all parameters are shown in Fig. 2. The ROX index is the best predictor of HFNC outcome with the highest discriminating capacity for HFNC success based on AUROC (AUROC 0.815, 0.784, 0.853 at 1, 2, and 6 h, respectively) (Table 3). In contrast, the HACOR scale showed an even lower discriminating ability to differentiate HFNC failure than respiratory rate with lower AUROC at all three timepoints (AUROC 0.733, 0.690, 0.702 at 1, 2, and 6 h, respectively). Also, the ROX index yielded the highest discriminating capacity than the other parameters in both the subgroup with and without pneumonia (Table S1). Moreover, the ROX index was a better predictor of HFNC success than the HACOR scale based on the logistic regression analyses. The ROX indices at all three timepoints were significant and independent predictors of HFNC success in all univariate and multivariate logistic regression models. In contrast, the HACOR scale failed to yield statistically significant associations with the outcome in some models, also with a generally lower strength of association in all models (Table 4). As for pulse rate, although it was generally higher in the failure group at all timepoints, its association with HFNC success was not as strong as those of respiratory rate and the ROX index (Tables 2 and 4).

**Table 3:** Area under the receiver operating characteristic curve (AUROC) of the parameters at 1, 2, and 6 h in predicting high-flow nasal cannula success or failure

Parameter	AUROC	*p*-value	95% confidence interval	
			**Lower bound**	**Upper bound**
Prediction of HFNC success				
ROX at 1 h	0.815	< 0.001	0.690	0.940
ROX at 2 h	0.784	< 0.001	0.629	0.939
ROX at 6 h	0.853	< 0.001	0.756	0.950
SF at 1 h	0.708	0.004	0.565	0.850
SF at 2 h	0.662	0.049	0.500	0.824
SF at 6 h	0.764	< 0.001	0.638	0.889
Prediction of HFNC failure				
HACOR at 1 h	0.733	0.003	0.578	0.887
HACOR at 2 h	0.690	0.036	0.512	0.868
HACOR at 6 h	0.702	0.032	0.517	0.886
RR at 1 h	0.773	< 0.001	0.630	0.915
RR at 2 h	0.754	0.001	0.599	0.908
RR at 6 h	0.842	< 0.001	0.722	0.963

*Abbreviations*: *HFNC* High-flow nasal cannula, *SF* Pulse oximetry/fraction of inspired oxygen ratio, *RR* Respiratory rate

**Table 4:** Univariate and multivariate analyses of potential factors predicting high-flow nasal cannula success

Variable	Univariate OR (95% CI; *p*-value)	Multivariate OR (95% CI; *p*-value)
ROX at 1 h	1.41 (1.05–1.87; *p* = 0.020)	1.49 (1.09–2.05; *p* = 0.014)
ROX at 2 h	1.38 (1.07–1.79; *p* = 0.013)	1.43 (1.09–1.89; *p* = 0.010)
ROX at 6 h	1.27 (1.001–1.61; *p* = 0.049)	1.34 (1.02–1.76; *p* = 0.036)
HACOR at 1 h	0.81 (0.66–0.99; *p* = 0.043)	0.77 (0.62–0.96; *p* = 0.021)
HACOR at 2 h	0.83 (0.68–1.01; *p* = 0.066)	0.81 (0.66–0.99; *p* = 0.046)
HACOR at 6 h	0.83 (0.65–1.06; *p* = 0.132)	0.79 (0.61–1.03; *p* = 0.083)
Respiratory rate at 1 h	0.84 (0.75–0.91; *p* = 0.002)	0.84 (0.75–0.95; *p* = 0.004)
Respiratory rate at 2 h	0.81 (0.71–0.93; *p* = 0.002)	0.80 (0.68–0.93; *p* = 0.004)
Respiratory rate at 6 h	0.74 (0.62–0.89; *p* = 0.001)	0.74 (0.60–0.91; *p* = 0.004)
Pulse rate at 1 h	0.97 (0.94–0.99; *p* = 0.042)	0.97 (0.95–1.00; *p* = 0.075)
Pulse rate at 2 h	0.98 (0.95–1.00; *p* = 0.064)	0.98 (0.95–1.01; *p* = 0.100)
Pulse rate at 6 h	0.95 (0.91–0.98; *p* = 0.005)	0.95 (0.92–0.99; *p* = 0.021)

The multivariate logistic regression models adjusted for age, sex, and underlying respiratory tract disease. Each multivariate model only employed one predictive score at one timepoint to avoid multicollinearity

*Abbreviations*: *OR* Odds ratio, *CI* Confidence interval

**Fig. 3:**
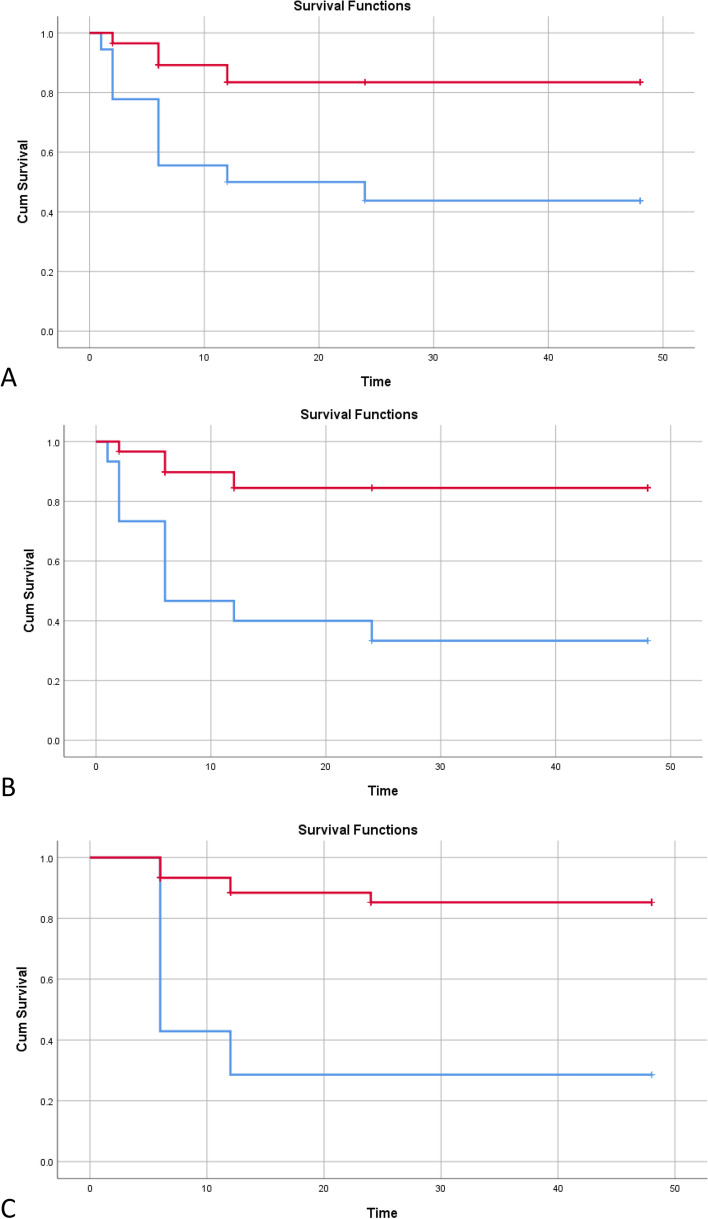
Survival function for high-flow nasal cannula failure of patients with the ROX index lower than 4.88 (blue line) compared to those with the index equal to or higher than 4.88 (red line) at 1 h (**A**), 2 h (**B**), and 6 h (**C**). The *p*-value for log rank test comparing survival functions between the groups at the ROX index cut-point of 4.88 = 0.001 (**A**) and < 0.001 (**B** and **C**)

We further evaluated the diagnostic accuracy indices of the ROX index, the best predictive parameter, based on the recommended cut-point of 4.88 and the optimal cut-point from our data of 4.75. The results are shown in Table 5. The highest sensitivity (94.23%) and NPV (76.92%) were achieved with the ROX index > 4.75 measured at 2 h, while the highest specificity (61.11%) and PPV (88.33%) were seen when analyzing the 6-h ROX index at either cut-points. Similarly, the ROX index at 6 h had the highest accuracy in predicting HFNC success. Additionally, patients with the ROX index of less than 4.88 had significantly lower survival function for HFNC failure based on the log-rank test compared to those with higher ROX at all three timepoints (log-rank *p*-value = 0.001, < 0.001, and < 0.0001 at 1, 2, and 6 h, respectively; Fig. 3).

**Table 5:** Diagnostic indices of the ROX index at the recommended (4.88) and optimal (4.75) cut-points

Time	ROX index	Sensitivity (%)	Specificity (%)	PPV (%)	NPV (%)	Accuracy	LR +	LR-
1 h	> 4.88	84.62	43.48	77.19	55.56	72.0	1.50	0.35
	> 4.75	90.38	43.48	78.33	66.67	76.0	1.60	0.22
2 h	> 4.88	90.38	43.48	78.33	66.67	76.0	1.60	0.22
	> 4.75	94.23	43.48	79.03	76.92	78.67	1.67	0.13
6 h	> 4.88	92.98	61.11	88.33	73.33	85.33	2.39	0.11
	> 4.75	92.98	61.11	88.33	73.33	85.33	2.39	0.11

*Abbreviations*: *PPV* Positive predictive value, *NPV* Negative predictive value, *LR* Likelihood ratio

## Discussion

To the best of our knowledge, this study was the first to directly compare the prognostic utility of the ROX index and the HACOR scale for undifferentiated AHRF in the ED setting. It was found that the ROX index was the best predictor of HFNC outcome since it could yield the highest discriminating ability for HFNC success. The index performed better once evaluated at 6 h post-HFNC application with the highest AUROC and most diagnostic indices higher than those measured at 1 or 2 h. In contrast, the HACOR scale was not a good predictor of HFNC failure, with the lowest discriminating ability compared to the other parameters.

HFNC has been increasingly used as the initial respiratory and oxygenation support measure in the ED. It has been shown to provide many physiologic benefits and may reduce adverse outcomes, such as mechanical ventilation rate [7–9, 30]. However, patients who fail HFNC treatment may still end up requiring invasive ventilation and a delay in such a process may lead to mortality [11, 12]. Therefore, appropriate and close observation with effective and efficient tools is mandatory to prevent these unfavorable consequences. The ROX index, one of the most widely validated prognostic indices for HFNC, was proposed for such purpose [17]. It has been proved as an accurate tool in predicting HFNC success in many studies, most of which were conducted in patients with pneumonia in the ICU setting [17, 18], whereas studies in the ED, whereby patient characteristics may be different and the cause of AHRF is usually unknown, are limited. Consequently, the present study has added to the current body of evidence that the ROX index is also an accurate predictor of HFNC success in the ED setting. Its AUROC was even slightly higher than those reported in some other external validation studies, and it could also yield favorable and comparable diagnostic accuracy indices at either of the cut-points studied [17, 18, 22]. The recommended cut-point of 4.88 from the literature [17] was feasible in our setting with almost as high diagnostic accuracy indices as the optimal cut-point from the data and also with highly significant log-rank tests. Additionally, we found that its prognostic utility seems to increase with time of HFNC treatment, similar to the ROX development study [18], which may have important clinical implications if the index measured at earlier timepoints are to be employed for clinical use in the ED as their lower predictive ability should be kept under consideration.

As for the HACOR scale, the present study found that it was the worst predictor of HFNC outcome among all the parameters studied. Its AUROC was largely smaller than those of the ROX index at all timepoints. One of the reasons why the HACOR scale was not a good predictor of HFNC failure was its relatively low weight anchored towards pulse rate, another known strong predictor of HFNC outcomes [28, 29]. However, this result was discordant with the only study that performed a pairwise comparison between the two parameters [22]. Despite similar AUROC of the HACOR scale between the present study and that previous study by Valencia et al. [22], they reported comparable discriminating capacity of the two parameters while we found the ROX index to be highly superior. This discordance could have most likely been due to different study population since they only enrolled patients with acute respiratory failure due to COVID-19 pneumonia in the ICU [22]. Another study by Magdy et al. [20] also evaluated the utility of the HACOR scale in predicting HFNC failure in the ICU and found that the HACOR scale could yield very high AUROC and diagnostic indices, contrary to our findings. The difference could have also been due to different study population. Most of the patients included in their study had pneumonia, while a largely higher proportion of our participants had cardiogenic pulmonary edema. In fact, in our subgroup analysis, we found that the AUROC of the ROX and the HACOR scale were more comparable in the pneumonia subgroup, which was concordant with those previous studies conducted in a similar patient population. Regardless, it would be interesting to explore the prognostic utility of these parameters in populations other than pneumonia patients, such as those with cardiogenic pulmonary edema, the most common second leading cause of AHRF [31]. Also, further studies should focus more on the potential benefit of HFNC in this population since we found a trend towards higher HFNC success in patients with AHRF than in those with pneumonia.

From the present study, it is appropriate to conclude that the ROX index is superior to the HACOR scale in predicting HFNC outcomes for undifferentiated AHRF in the ED setting not only because of the overall statistical indices but also due to the fact that the ROX index is easier to calculate at bedside and does not require arterial gas analysis, thereby being more efficient to use in the ED.

The present study also had limitations. First, it was a single-center study, possibly limiting the generalizability of the study results. Second, the sample size was smaller than intended since the study had to be discontinued due to the first COVID-19 outbreak, causing most analyses to be underpowered. The small sample size resulted in wide standard errors of the AUROCs, causing the differences to be most likely non-statistically significant. Despite such an issue, we could still demonstrate a substantial difference between the point estimates of the AUROC of the two main study parameters. Therefore, we can be quite certain of the relative difference in discrimination capacity between the two parameters, although the exact effect estimates may not be accurate due to random error. Third, despite the studying having a clearly defined termination and escalation criteria that did not include the ROX index, there could still have been some physicians who determined their treatment decisions based on the ROX index, a known widely used parameter, thus possibly causing a falsely high association and predictive ability for the ROX index. Fourth, although it would have been interesting had the parameters been followed longer than 6 h with the increasing trend of prognostic utility observed, the relevance of such results to the ED setting may be limited. Nevertheless, future multicenter studies with an adequate sample size including patients of various etiologies of AHRF should be performed to confirm the present study’s results and explore further prognostic utility of these parameters.

## Conclusion

The ROX index was better than the HACOR scale at predicting HFNC outcome (success/failure) in patients with AHRF of undifferentiated origin in the ED setting. It has superior prognostic utility with less complexity and higher efficiency to be employed at bedside compared to the HACOR scale. Therefore, the ROX index is a more appropriate tool to guide further management and potential escalation therapy for AHRF patients with HFNC therapy initiated early in the ED.

## Supplementary Information

**Additional file 1: Table S1.** Area under the receiver operating characteristics curve (AUROC) of the parameters at 1, 2, and 6 hours in predicting high-flow nasal cannula success or failure in a subgroup with pneumonia and without pneumonia.

## Data Availability

The dataset is not available but can be requested from the corresponding author.

## Abbreviations

AHRF: Acute hypoxemic respiratory failure
ED: Emergency department
HFNC: High-flow nasal cannula
FiO_2_: Fraction of inspired oxygen
ICU: Intensive care unit
SOFA: Sequential organ assessment
SAP II: Simplified acute physiology II
APACHE II: Acute physiology and chronic health evaluation II
ROX: Respiratory rate Oxygenation
SpO_2_: Oxygen saturation
HACOR: Heart rate, acidosis, consciousness, oxygenation, and respiratory rate
GCS: Glasgow Coma Scale
NIV: Non-invasive ventilation
ROC: Receiver operating characteristic
AUROC: Area under the curve of the receiver operator characteristic curves
LR +: Positive likelihood ratio
LR-: Negative likelihood ration
NPV: Negative predictive value
PPV: Positive predictive value
