# Supplementary material for: ROX index versus HACOR scale in predicting success and failure of high-flow nasal cannula in the emergency department for patients with acute hypoxemic respiratory failure: a prospective observational study
Source: Int J Emerg Med. 2023 Jan 10;16:3. doi: 10.1186/s12245-023-00477-1 (PMC9830606; doi:10.1186/s12245-023-00477-1)
Supplement: Supplementary file 1 — Additional file 1: Table S1. Area under the receiver operating characteristics curve (AUROC) of the parameters at 1, 2, and 6 hours in predicting high-flow nasal cannula success or failure in a subgroup with pneumonia and without pneumonia. [file 12245_2023_477_MOESM1_ESM.pdf]

Table S1. Area under the receiver operating characteristics curve (AUROC) of the parameters at 1, 2, and 6 hours in predicting high-flow nasal cannula success or failure in a subgroup with pneumonia and without pneumonia

Parameter	Pneumonia subgroup (n=30)		Non-pneumonia subgroup (n=45)	
	AUROC (95% CI)	p-value	AUROC (95% CI)	p-value
Prediction of HFNC success				
ROX at 1 hour	0.845 (0.693-0.997)	0.002	0.662 (0.417-0.907)	0.154
ROX at 2 hour	0.839 (0.673-1.000)	0.004	0.650 (0.400-0.901)	0.186
ROX at 6 hour	0.850 (0.705-0.995)	0.007	0.811 (0.671-0.952)	0.026
SF at 1 hour	0.703 (0.514-0.891)	0.075	0.628 (0.398-0.859)	0.259
SF at 2 hour	0.681 (0.476-0.885)	0.126	0.635 (0.423-0.847)	0.235
SF at 6 hour	0.729 (0.530-0.927)	0.077	0.749 (0.671-0.952)	0.026
Prediction of HFNC failure				
HACOR at 1 hour	0.760 (0.582-0.938)	0.022	0.595 (0.350-0.839)	0.406
HACOR at 2 hour	0.775 (0.587-0.963)	0.020	0.561 (0.301-0.821)	0.593
HACOR at 6 hour	0.764 (0.574-0.955)	0.041	0.537 (0.209-0.865)	0.790
RR at 1 hour	0.748 (0.562-0.933)	0.029	0.677 (0.476-0.879)	0.119
RR at 2 hour	0.736 (0.511-0.961)	0.045	0.669 (0.449-0.889)	0.138
RR at 6 hour	0.861 (0.716-1.000)	0.005	0.791 (0.569-1.000)	0.037

Abbreviation: HFNC, high-flow nasal cannula; SF, pulse oximetry/fraction of inspired oxygen ratio; RR, respiratory rate
